# Darwinian Evolution on a Chip

**DOI:** 10.1371/journal.pbio.0060085

**Published:** 2008-04-08

**Authors:** Brian M Paegel, Gerald F Joyce

**Affiliations:** 1 Departments of Chemistry and Molecular Biology, The Scripps Research Institute, La Jolla, California, United States of America; 2 The Skaggs Institute for Chemical Biology, The Scripps Research Institute, La Jolla, California, United States of America; University of Wisconsin Madison, United States of America

## Abstract

Computer control of Darwinian evolution has been demonstrated by propagating a population of RNA enzymes in a microfluidic device. The RNA population was challenged to catalyze the ligation of an oligonucleotide substrate under conditions of progressively lower substrate concentrations. A microchip-based serial dilution circuit automated an exponential growth phase followed by a 10-fold dilution, which was repeated for 500 log-growth iterations. Evolution was observed in real time as the population adapted and achieved progressively faster growth rates over time. The final evolved enzyme contained a set of 11 mutations that conferred a 90-fold improvement in substrate utilization, coinciding with the applied selective pressure. This system reduces evolution to a microfluidic algorithm, allowing the experimenter to observe and manipulate adaptation.

## Author Summary

The principles of Darwinian evolution are fundamental to understanding biological organization and have been applied to the development of functional molecules in the test tube. Laboratory evolution is greatly accelerated compared with natural evolution, but it usually requires substantial manipulation by the experimenter. Here we describe a system that relies on computer control and microfluidic chip technology to automate the directed evolution of functional molecules, subject to precisely defined parameters. We used a population of billions of RNA enzymes with RNA-joining activity, which were challenged to react in the presence of progressively lower concentrations of substrate. The enzymes that did react were amplified to produce progeny, which were challenged similarly. Whenever the population size reached a predetermined threshold, chip-based operations were executed to isolate a fraction of the population and mix it with fresh reagents. These steps were repeated automatically for 500 iterations of 10-fold exponential growth followed by 10-fold dilution. We observed evolution in real time as the population adapted to the imposed selection constraints and achieved progressively faster growth rates over time. Our microfluidic system allows us to perform Darwinian evolution experiments in much the same way that one would execute a computer program.

## Abstract 3

Darwinian evolution has been captured as a microchip-based operation, allowing the experimenter to observe and control in real time the adaptation of a population of RNA enzymes.

## Introduction

The scientific community will soon celebrate the 200th anniversary of the birth of Charles Darwin and the 150th anniversary of the publication of his seminal work *On the Origin of Species by Means of Natural Selection* [1]. The principles of Darwinian evolution are fundamental to understanding biological organization at the level of populations of organisms and for explaining the development of biological genomes and macromolecular function. Darwinian evolution also has become a chemical tool for discovering and optimizing functional macromolecules in the test tube (for recent reviews, see [2–5]). Laboratory evolution is greatly accelerated compared with natural evolution but requires substantial manipulation by the experimenter, which is imprecise, time consuming, and usually performed in an ad hoc manner.

Many laboratory procedures have been miniaturized using microfluidic technology, which reduces cost and increases precision over manual methods [6]. In the present study, a system is described that relies on computer control and microfluidic chip technology to automate the directed evolution of functional molecules, a process that is subject to precisely defined parameters. A population of billions of RNA enzymes with RNA ligase activity was made to evolve continuously, with real-time monitoring of the population size and fitness. Whenever the population size reached a predetermined threshold, chip-based operations were executed to isolate a fraction of the population and mix it with a fresh supply of reagents. These steps repeated automatically as the population adapted to the imposed selection constraints within a period of several hours.

The RNA enzyme that was chosen for this study is a descendant of the class I RNA ligase, first described by Bartel and Szostak [7]. It is one of only two RNA enzymes that have been made to undergo continuous in vitro evolution [8,9], a process in which all of the components necessary for evolution are contained within a common reaction vessel. The enzyme is challenged to ligate a promoter-containing oligonucleotide substrate to itself by catalyzing nucleophilic attack of the 3′-hydroxyl of the substrate on the 5′-triphosphate of the enzyme. The reaction mixture also contains two polymerase enzymes (reverse transcriptase and T7 RNA polymerase) that amplify any RNA molecules that have acquired the promoter sequence as a consequence of RNA-catalyzed ligation. Multiple copies of progeny RNA are generated, which then can enter another cycle of reaction and selective amplification. In a population of variant RNA enzymes, those that react most efficiently grow to dominate the population in the competition for limited chemical resources. A serial transfer or serial dilution protocol is used to refresh periodically the supply of reagents, allowing the evolution process to continue indefinitely.

## Results

**Figure 1:**
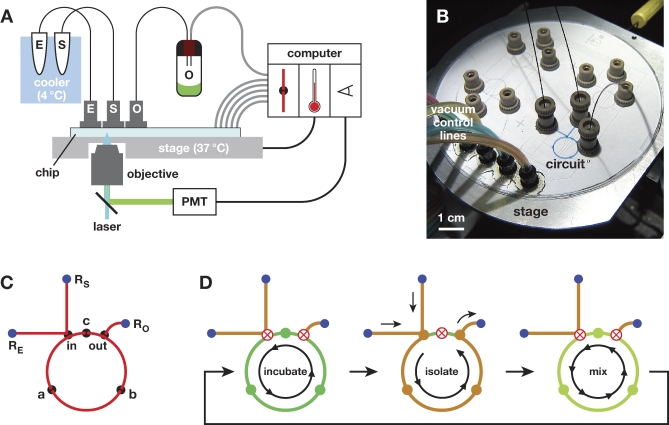
Microfluidic Evolution System (A) The evolution chip is mounted on a temperature-controlled stage. Solutions containing polymerase enzymes (*E*) and mono- and oligonucleotide components (*S*) are delivered to the chip via capillary tubing and output to a pressure-controlled collection vial (*O*). A microscope objective is used to focus laser excitation (λ_ex_ = 490 nm) on the microfluidic channel and to gather fluorescence (λ_em_ = 535 nm), which is detected with a confocal PMT. Valve actuation and fluid flow are controlled by six independent vacuum lines. (B) The microfluidic device is shown with the active circuit filled with blue dye. (C) The serial dilution circuit consists of a mixing loop with fluid flow channels (red), fluid access reservoirs (blue), and control valves (black). Fluid flow around the loop is controlled by three two-way valves (*a*, *b*, and *c*). Fluid access to the loop from the input reservoirs (*R_E_* and *R_S_*) and to the output reservoir (*R_O_*) is controlled by bus valves (*in* and *out*). The bus valves allow access when open, and prevent access while preserving fluidic continuity within the loop when closed. (D) During operation of the circuit, the expanding RNA population is incubated while undergoing slow cyclic mixing until the fluorescence reaches a pre-determined threshold. Then an aliquot of the population is isolated between valves *in* and *out* as fresh solutions of *E* and *S* are drawn into the loop and spent materials are delivered to the collection vial. Finally, the loop is sealed by closing valves *in* and *out*, and the aliquot is mixed with the fresh solutions by rapid serial actuation of valves *a*, *b*, and *c*. Open valves are indicated by filled circles; closed valves are indicated by a red X.

The chip-based evolution system consists of a microfluidic device mounted on a temperature-controlled stage and monitored by an inverted confocal fluorescence microscope (Figure 1A and 1B). A laptop computer controls the actuation of valves on the chip and the acquisition and processing of fluorescence data indicate the concentration of RNA enzymes within the microfluidic circuit. The circuit consists of a mixing loop (1 cm diameter, 400 nl volume), with three in-line valves for mixing and two bus valves that control the input of fresh reagents and the output of spent reaction materials (Figure 1C). This device can perform serial dilutions in a rapid and precise manner [10]. Each iteration of events on the chip entails an incubation step with slow mixing, an isolation step in which one-tenth of the reaction mixture is retained in part of the circuit while fresh reaction materials are drawn into the remainder of the circuit, and a rapid mixing step in which the isolated aliquot is combined with the fresh reagents (Figure 1D). The reaction mixture contains thiazole orange, which intercalates into nucleic acids and upon laser excitation gives a characteristic fluorescent signal. When the fluorescence reaches a predetermined threshold, correlating with a 10-fold increase in the concentration of RNA, the computer initiates an automated 10-fold dilution.

Continuous evolution on the chip was initiated with randomized variants of the “B16–19” form of the class I ligase RNA enzyme [11]. Like other forms of the class I ligase, this enzyme has an impressive catalytic rate of 20 ± 2 min^−1^, but a somewhat poor Michaelis constant (*K*_m_) of 35 ± 8 μM (measured in the presence of 10 mM MgCl_2_ and 50 mM KCl at pH 7.5 and 37 °C). Random mutations were introduced throughout the molecule at a frequency of ∼0.7% per nucleotide position using a mutagenic PCR procedure [12], followed by in vitro transcription. A starting population of 2 × 10^9^ variants was introduced to the chip and challenged to catalyze RNA ligation under conditions of progressively reduced substrate concentrations. This selection pressure was expected to favor individuals with an improved *K*_m_. At the outset, the substrate concentration was 1 μM, causing the starting B16–19 enzyme to operate with an observed rate of only 0.6 min^−1^. Any individuals with an improved *K*_m_ would operate at a faster rate, and therefore would undergo more rapid amplification and grow to dominate the population. As the evolving population adapted to the reduced substrate concentration, the concentration was reduced further, eventually reaching just 0.05 μM.

**Figure 2:**
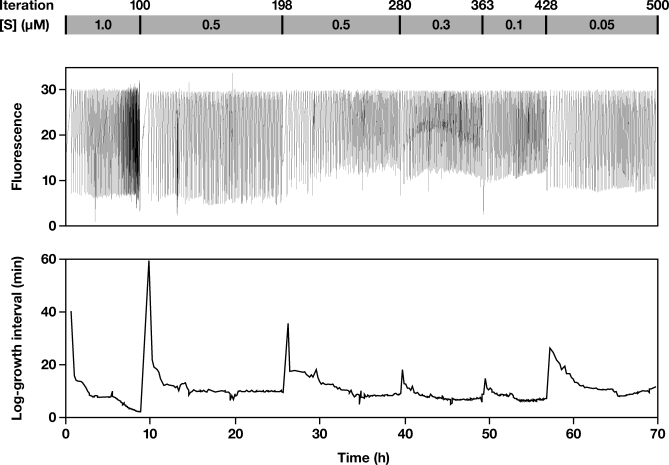
Chip-Based Evolution RNA enzymes were evolved over the course of 500 logs of selective amplification. The population was monitored in real time based on fluorescence intensity (middle). After each log of growth, a 10-fold dilution was executed by automated microfluidic manipulation. The substrate concentration was reduced periodically to maintain selective pressure on the evolving population (top). Before each reduction in substrate concentration, sample was removed from the circuit, mutagenized, and reintroduced to the circuit. The interval between successive dilutions was recorded as a function of time (bottom).

The course of evolution was monitored continuously based on fluorescence, tracking the time needed to achieve 10-fold overall amplification of the RNA population (Figure 2). The log-linear growth rate of the starting population during the first 40 min was used to set the fluorescence threshold for the circuit, before executing the first 10-fold dilution. One hundred iterations of log-growth and dilution were executed in the presence of 1 μM substrate. The instantaneous fitness of the population was reflected by the interval between successive dilutions, which decreased monotonically over the first 100 log-growth iterations.

Materials collected from iterations 95–100 were pooled, subjected to mutagenic PCR, and reintroduced to the chip, but now the substrate concentration was reduced to 0.5 μM. This resulted in a temporary decrease in fitness (increased dilution interval), but the population quickly adapted, achieving 10-fold growth every 10 min by iteration 198. At that point, and at iterations 280, 363, and 428, materials again were collected, mutagenized, and returned to the chip. The substrate concentration was reduced to 0.3 μM at iteration 280, to 0.1 μM at iteration 363, and finally to 0.05 μM at iteration 428.

**Figure 3:**
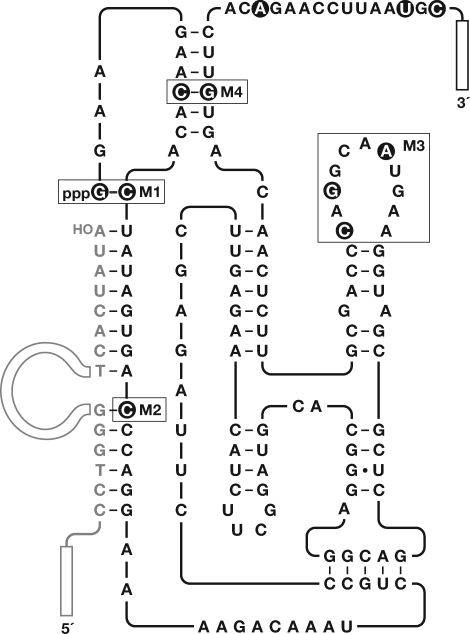
Evolution of Genotype The diagram shows the sequence and secondary structure of the final evolved ligase enzyme. The oligonucleotide substrate is shown in gray, specifying those residues that bind to the template region of the RNA enzyme. The primer binding sites at the 3′ end of the enzyme and 5′ end of the substrate are indicated by open rectangles. Mutations that were present in all sequenced clones are highlighted by black circles. Critical mutations, designated M1, M2, M3, and M4, are indicated by boxes.

After 500 iterations of log-growth and dilution (70 h on the chip), the evolution process was deemed complete. Individuals were cloned from the population at iterations 198 and 500 and sequenced. At iteration 500, all of the sequenced clones contained 11 mutations, which could be divided into four subgroups (M1, M2, M3, and M4) based on their relationship to the known secondary structure of the class I ligase (Figure 3). The M1 mutations occur immediately on the 3′ side of the ligation junction, replacing the pppA•U pair by a pppG•C pair. These mutations restore the pppGpG transcription initiation sequence that is preferred by T7 RNA polymerase [13], while maintaining Watson-Crick pairing of the 5′-terminal guanosine. The M2 mutation is a C insertion that appears to extend the template region of the enzyme so that it binds six additional nucleotides in the upstream portion of the substrate. The M3 mutations (one transition, one transversion, and one insertion) all occur within a hairpin loop that is thought to lie in close proximity to the template region, based on modeling of the three-dimensional structure of the class I ligase [14]. The M4 mutations change a U•A pair to a G•C pair within a stem adjacent to the ligation junction.

**Figure 4:**
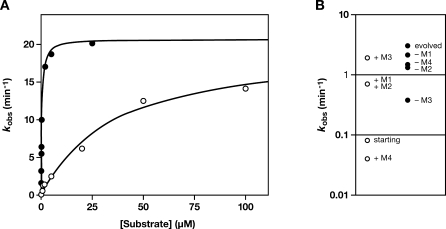
Evolution of Phenotype Catalytic activity was measured for the starting (open circles) and final evolved (filled circles) enzymes. (A) The observed rate constant, *k*_obs_, was determined for various concentrations of substrate and fit to the Michaelis-Menten equation. (B) Values for *k*_obs_ were obtained in the presence of 0.1 μM substrate for variants of the starting enzyme that contained each of the four critical mutations (left) and for variants of the evolved enzyme that lacked each of these mutations (right).

A representative clone that contained all 11 conserved mutations, as well as three mutations near the 3′ end, was examined with regard to its catalytic properties. It exhibited a *k*_cat_ of 21 ± 0.8 min^−1^, which is nearly identical to that of the starting enzyme, and a *K*_m_ of 0.4 ± 0.05 μM, which corresponds to a 90-fold improvement (Figure 4A). The fact that only *K*_m_ improved reflects the selective pressure that had been placed on the population. The starting B16–19 form of the enzyme was evolved to operate in the presence of 5 μM substrate [8,11], and this concentration was reduced by 100-fold during the course of 500 logs of on-chip evolution. Thus, the improvement in *K*_m_ closely parallels the degree of selective pressure that was applied.

To assess the individual contribution of the M1–M4 mutations, each was added to the starting enzyme and each was removed from the final evolved enzyme. The observed reaction rate in the presence of 0.1 μM substrate was determined for each construct (Figure 4B). Adding the M1 or M2 mutations to the starting enzyme caused a 9-fold increase in the observed rate, whereas adding the M3 mutations caused a 24-fold increase. Surprisingly, adding the M4 mutations caused a 2-fold decrease. Reverting the M1, M2, or M4 mutations within the evolved enzyme caused a 2- to 3-fold decrease in the observed rate, while reverting the M3 mutations caused a 10-fold decrease. Thus the M1, M2, and M3 mutations appear to exhibit independent effects, whereas the M4 mutation only confers selective advantage on the background of the other mutations. Clearly, the M3 mutations are the most significant. When they are added to the starting enzyme, there is no change in *k*_cat_ and there is a 10-fold improvement in *K*_m_. Conversely, when the M3 mutations are removed from the evolved enzyme, there is no change in *k*_cat_ and there is a 10-fold worsening of *K*_m_ (Figure S1).

Returning to iteration 198, when the population had only been challenged to adapt to 0.5 μM substrate, none of 20 cloned sequences contained the M4 mutations. However, all of the cloned sequences contained the M1 and M2 mutations. The M3 mutations were beginning to appear at that time, with all 20 clones containing the G insertion (Figure 3), but only eight containing the A→C transversion and only two containing the G→A transition. The insertion and transversion mutations are intriguing because they result in the sequence 5′-GACCCAG-3′ (mutations underlined), which is identical to the sequence 5′-GACCCAG-3′ (M2 mutation underlined) that occurs within the extended template region of the enzyme. It is possible that one or both of these regions engages in pairing interactions with the substrate.

Site-directed mutagenesis studies were carried out on the final evolved enzyme to examine potential enzyme–substrate interactions enabled by the M2 and M3 mutations. An alternative substrate was prepared, leaving unchanged the eight nucleotides that are complementary to the original template region of the enzyme, but changing the six nucleotides that are complementary to the extended template region (Figure 3). No activity was observed with this substrate. Activity was largely restored, however, by introducing compensatory mutations within the enzyme that reestablished Watson-Crick complementarity with the substrate (Figure S2). An enzyme containing these six mutations could not react with the original substrate, also consistent with the requirement for complementarity in this region. Interestingly, the region of the M3 mutations could compensate for a partial mismatch within the extended template region. If just four of the six nucleotides in the extended template region were mutated, activity was retained so long as the M3 region was left unchanged. If the M3 region also was mutated, then there was no detectable activity (Figure S2). Thus the region of the M3 mutations appears to assist the extended template region in recognizing the substrate.

## Discussion

The constellation of mutations that arose over the course of evolution could not have been anticipated, especially the triple mutations within the M3 region that had the most dramatic effect on *K*_m_ and the paired M4 mutations that only had benefit when combined with the other mutations. It is not surprising that these more complex traits arose later in the evolution process. The snapshot of the evolving population that was obtained at iteration 198 revealed intermediate forms, with two advantageous traits (M1 and M2) already acquired and the acquisition of a third (M3) still in progress. If sequence analyses were carried out at more frequent intervals during the 500 iterations of log-growth and dilution, it would provide a more detailed picture of the ebb and flow of genetic traits. This genetic information could be correlated with measurements of the catalytic behavior and growth rate for each of the cloned individuals. Methods exist for microfluidic-based clonal isolation and amplification [15], DNA sequencing [16], and analysis of enzyme kinetics [17], raising the possibility that Darwinian evolution and analysis of the evolving population could be carried out in an integrated microfluidic format.

It is possible that further evolution on the chip, carried out in the presence of even lower concentrations of substrate, would lead to further improvement in *K*_m_. Ultimately, however, this improvement would be limited by three constraints: (1) reduced size of the evolving population when operating at very low substrate concentrations, yet maintaining conditions of substrate excess; (2) technical limitations in fluorescence monitoring of sub-nanomolar concentrations of RNA; and (3) intrinsic limitations on the ability of RNA to catalyze templated RNA ligation. With regard to the latter, the catalytic efficiency, *k*_cat_/*K*_m_, of the final evolved enzyme is 5 × 10^7^ M^−1^ min^−1^. This is close to the rate of association of two complementary oligonucleotides, which is ∼10^9^ M^−1^ min^−1^ under similar reaction conditions [18–20]. If the catalytic rate of the RNA enzyme remained about 20 min^−1^, then it would not be possible for *K*_m_ to improve to better than about 20 nM (a 20-fold improvement compared with the present value), unless the enzyme evolved a means to bind the substrate faster than the inherent rate of duplex formation.

The microfluidic system could be used to obtain RNA enzymes with a variety of phenotypes, including those that have been obtained by conventional in vitro evolution methods. Microfluidic technology also might be used to evolve proteins, viruses, and even cellular organisms. Bacterial populations have been maintained in a microfluidic bioreactor [21], and that system could, in principle, be used to conduct evolution experiments. However, when evolution is carried out at the level of molecules rather than cells, one has ready access to the genotype and phenotype of individuals in the population throughout the course of their evolutionary history. Such access makes it possible to witness evolutionary adaptation and to determine the particular genetic mutations and corresponding phenotypic changes that are responsible for that adaptation.

The chief advantages of chip-based evolution are its precision and ease of operation. The runtime parameters for evolution are established at the outset and are enforced precisely throughout the course of an experiment. The continuous stream of real-time data provides a high-resolution record of an evolutionary trajectory, which can be obtained as a function of population size, population heterogeneity, growth conditions, and the availability of limiting resources. Each microchip contains multiple microfluidic circuits that can be addressed independently, and the chip as a whole can be produced at nominal cost. Thus, Darwinian evolution becomes commoditized, allowing one to perform many evolution experiments with little more difficulty than the execution of a computer program.

## Materials and Methods

### Starting pool of RNA.

Plasmid DNA encoding the B16–19 form of the class I RNA ligase [11] was PCR amplified using a primer that converted the 3′-terminal nucleotides of the enzyme to 5′-ACGAGCAUGGAGGGACU-3′, so as to bind a different cDNA primer. The PCR product was purified by agarose gel electrophoresis, then subject to error-prone PCR, which introduced random mutations at a frequency of ∼0.7% per nucleotide position [12]. The resulting DNA was transcribed in vitro to generate the starting pool of RNA, which was purified by denaturing polyacrylamide gel electrophoresis (PAGE) and desalted on Sephadex G-25. The microfluidic circuit was primed with a solution of 100 nM starting pool RNA, 15 mM MgCl_2_, 50 mM KCl, 50 mM EPPS (pH 7.5), 4 mM DTT, 0.1% IGEPAL-CA630 (used to reduce surface tension), and 0.1 μM fluorescein dye (used as a tracer).

### Microchip control system.

A microfluidic serial dilution circuit [10] with a carryover fraction of 0.1 was used for the on-chip continuous evolution experiments. The microfabrication process [22] and membrane valve technology [23] have been described previously. The circuit design was modified by splitting the input channel into separate inputs for the polymerase enzymes (*E*) and mono- and oligonucleotide components (*S*), converging at the deflection chamber for the *in* bus valve (Figure 1C). Fluidic and vacuum channel features were etched into separate glass wafers to a depth of 50 μm. The fluidic and vacuum channels had widths of 300 μm and 55 μm, respectively. The mixing loop had a diameter of 1 cm and a volume of 400 nl. The microfluidic device was vacuum-chucked to an aluminum stage fitted with an annular thin-film heater (5548, Minco) and K-type thermocouple probe, controlled by a PID temperature controller (CNi32, Omega Engineering). Data acquisition, pneumatic control, and temperature control were handled by a laptop computer equipped with a NI6715 data acquisition card and software written in-house (LabVIEW, National Instruments).

Computer-controlled pneumatic actuation of valves on the chip was accomplished with a solenoid valve array (HV011, Humphrey). PEEK capillary tubing (25 μm inner diameter, Upchurch Scientific) was used to deliver reagents and collect samples from the device, interfaced with fluid access reservoirs using finger-tight capillary tubing fittings (N-123s, Upchurch Scientific). The sample collection line was pierced through the septum of a 2-ml silanized glass autosampler vial. An additional pneumatic control line fitted with a 24 gauge syringe was inserted through the septum and used to control depressurization of the sample collection vial.

Diode laser excitation (490 nm, Coherent) was coupled into the detection optical train using a dichroic long-pass mirror (505DRLP, Omega Optical) and focused on the fluidic channel using a microscope objective (40×, 0.6 NA, Newport). Fluorescence emission was collected by the same objective, spectrally filtered using a bandpass filter (535DF60, Omega Optical) and spatially filtered with a 100-μm pinhole prior to illuminating a PMT detector (H7827, Hamamatsu Photonics). Fluorescence data were acquired at 0.1 Hz and processed with a five-point averaging filter.

### Microfluidic programs.

Fluid handling on the chip was accomplished by three valve actuation programs: *prime*, *mix*, and *isolate*. The *prime* program consists of opening valves *a*, *b*, *c*, *in*, and *out*, then depressurizing the sample collection vial (Figure 1C). This draws reagent in through the *E* and *S* sample lines, flushing the entire circuit with reagent and depositing the waste into the collection vial. The *mix* program pumps fluid around the mixing loop by serially actuating valves *a*, *b*, and *c*. The wait time between valve actuations is 300 ms for slow mixing during incubation steps and 80 ms for rapid mixing following the introduction of fresh reagents (Figure 1D). The *isolate* program consists of opening valves *a*, *b*, *in*, and *out* and depressurizing the sample collection vial. This flushes reagent through the outside portion of the mixing loop containing *a* and *b*, while isolating an aliquot of material in the region containing *c* and bounded by *in* and *out*. Executing *isolate* followed by rapid *mix* results in 10-fold dilution of the carryover materials and constitutes one iteration of the continuous evolution process.

### Continuous evolution on chip.

Prior to use, each new circuit was flushed with a rinse solution containing 50 mM EPPS (pH 7.5) and 0.1% IGEPAL-CA630, executing *prime* for 10 s every min over a 60-min period. During this process, the stage temperature was stabilized at 38.5 °C to maintain 37 °C within the device (previously calibrated). Continuous evolution was initiated on the device by immersing both the *E* and *S* lines in the solution of starting pool RNA. The circuit was primed until the fluorescence intensity of the fluorescein tracer had stabilized. Then the *E* and *S* lines were immersed in the rinse solution, and the circuit executed a 60-s *isolate* program to flush the input lines and isolate an aliquot of the starting pool of RNA within the circuit.

The collection vial was repressurized, and the *E* line was immersed in a solution containing 20 U/μl Superscript II reverse transcriptase (Invitrogen), 5 U/μl T7 RNA polymerase, 0.001 U/μl yeast inorganic pyrophosphatase (Sigma-Aldrich), 15 mM MgCl_2_, 50 mM KCl, 50 mM EPPS (pH 7.5), 4 mM DTT, 0.1% IGEPAL-CA630, and 6 μM TO-PRO-1 (Invitrogen). The *S* line was immersed in a separate solution containing the oligonucleotide substrate, 5 μM cDNA primer having the sequence 5′-AGTCCCTCCATGCTCGT-3′, 4 mM each NTP, 0.4 mM each dNTP, 15 mM MgCl_2_, 50 mM KCl, 50 mM EPPS (pH 7.5), 4 mM DTT, 0.1% IGEPAL-CA630, and 6 μM TO-PRO-1. The oligonucleotide substrate, which was present at progressively lower concentrations during the course of evolution, had the sequence 5′-CCGAAGCCTGGGATCAATAATACGACTCAC**UAUA**-3′ (T7 RNA polymerase promoter sequence underlined; RNA residues in bold). Aqueous glycerol (50%) was added to the substrate-containing solution to match the viscosity of the polymerase-containing solution. Both solutions were thermoelectrically cooled to preserve the activity of the polymerase enzymes.

The collection vial was again depressurized to draw a 1:1 mixture of the substrate- and polymerase-containing solutions, which primed the input lines and filled the circuit in the region containing *a* and *b* and bounded by *in* and *out*. The circuit then was directed to execute rapid *mix* for 40 s, followed by slow *mix* during the incubation phase of the evolution procedure. The slow cyclic mixing prevented photobleaching of the fluorescent dye. The intercalating dye TO-PRO-1 was chosen for its superior enhanced fluorescence quantum yield upon binding double-stranded nucleic acids [24]. The background fluorescence prior to RNA amplification was typically ∼10 kCPS. The time required for 10-fold growth of the starting RNA enzyme in the presence of 1 μM substrate was determined to be 40 min. This established the incubation time for the first round and set the threshold for dilution at 30 kCPS. Subsequently, whenever the detector registered 30 kCPS, the circuit was directed to execute the steps of *isolate*, delivery of fresh reagents with rapid *mix*, and incubation with slow *mix*.

The population of RNA enzymes underwent continuous evolution on the chip until the time between successive log dilutions either decreased below 2 min or exhibited no further improvement. Materials from the last five iterations were collected in a fresh vial that contained 50 μl of 0.1 *N* NaOH. This mixture was incubated at 95 °C for 10 min to hydrolyze the RNA components, and the remaining DNA then was amplified by both standard PCR (as a control) and error-prone PCR. The primers for PCR amplification had the sequence 5′-AGTCCCTCCATGCTCGT-3′ and 5′-CCGAAGCCTGGGATCAATAA-3′. The sample collected after iteration 198 was mutagenized at a frequency of ∼10% per nucleotide position using a hypermutagenic PCR protocol [25]. Samples collected after iterations 280, 363, and 428 were mutagenized using standard error-prone PCR [12]. The PCR products were transcribed, and the resulting RNAs were purified by PAGE, desalted, and resuspended in a solution containing 15 mM MgCl_2_, 50 mM KCl, 50 mM EPPS (pH 7.5), 4 mM DTT, 0.1% IGEPAL-CA630, and 0.1 μM fluorescein, which was used to start the next set of iterations on the chip. The concentration of RNA in the start solution was 100 nM following iterations 198 and 280, 20 nM following iteration 363, and 10 nM following iteration 428, thereby maintaining the substrate in excess of the RNA enzyme throughout the evolution process.

### Analysis of individual RNA enzymes.

The PCR products obtained following iterations 198 and 500 were cloned and sequenced. An individual corresponding to the consensus sequence of 10 clones that were sequenced after iteration 500 was PCR amplified, transcribed in the presence of [α-^32^P]ATP, purified by PAGE, and desalted. Its catalytic activity was measured in the presence of 10 mM MgCl_2_, 50 mM KCl, and 50 mM EPPS (pH 7.5) at 37 °C, determining the observed rate constant in the presence of 10 nM enzyme and varying concentrations of substrate. Reactions were initiated by adding equal volumes of enzyme and substrate solutions, each containing all of the other reaction components and pre-equilibrated at 37 °C. Aliquots were taken at various times and quenched by the addition of 15 mM EDTA. For very short reaction times (<5 s), the reaction was carried out in a quench-flow apparatus (KinTek), using separate syringes to deliver the enzyme, substrate, and quench solutions.

The reaction products were separated by PAGE and quantitated using a PharosFX molecular imager (Bio-Rad). Biphasic kinetics were observed at all substrate concentrations. The overall maximum extent of the reaction was determined empirically by measuring the fraction reacted at 2- and 3-h time points. The data were fit to the equation: *F*(*t*) = *F*_max_ – *A*_1_e^–*k*1*t*^ – *A*_2_e^–*k*2*t*^, where *F*_max_ is the maximum extent, *A*_1_ and *k*_1_ are the amplitude and rate of the initial fast phase, and *A*_2_ and *k*_2_ are the amplitude and rate of the subsequent slow phase, respectively. The amplitude of the fast phase typically was 0.6–0.7 and the overall maximum extent typically was 0.9. A saturation plot was constructed by plotting *k*_1_ as a function of substrate concentration, and these data were fit to the Michaelis-Menten equation to determine *k*_cat_ and *K*_m_.

Variants of the starting and final evolved enzymes that contained different combinations of the four critical mutations were prepared by PCR amplification, using appropriate primers to introduce the desired mutations. The PCR products were transcribed in the presence of [α-^32^P]ATP, purified by PAGE, and desalted. The maximum extent of reaction and observed rate constant were determined in the presence of 0.1 μM substrate for each variant. In addition, a variant of the starting enzyme that contained the M3 mutations and a variant of the evolved enzyme that lacked the M3 mutations were subject to formal kinetic analysis, as described above.

## Supporting Information

Figure S1 Influence of Mutations on Catalytic Activity Catalytic activity of the starting enzyme with the M3 mutations added (open circles) and of the final evolved enzyme with the M3 mutations removed (filled circles). The observed rate constant, *k*_obs_, was determined for various concentrations of substrate and fit to the Michaelis-Menten equation. The starting enzyme with M3 mutations added exhibited a *k*_cat_ of 24.8 ± 1.4 min^−1^ and *K*_m_ of 2.4 ± 0.5 μM. The evolved enzyme with M3 mutations removed exhibited a *k*_cat_ of 24.0 ± 0.8 min^−1^ and *K*_m_ of 3.9 ± 0.4 μM.
(244 KB PDF)

Figure S2 Site-Directed Mutagenesis Studies Site-directed mutagenesis was carried out to analyze regions of the final evolved enzyme and the oligonucleotide substrate that appear to interact. The sequence of these regions prior to mutagenesis is shown in blue, and the mutations that were introduced are shown in red. Changes to the substrate alone are indicated at the left, changes to the extended template region of the enzyme and corresponding portion of the substrate are indicated at the bottom, and changes to the region of the M3 mutations are indicated at the right. Values for *k*_obs_ were obtained in the presence of 0.1 μM substrate, and are shown below the corresponding sequence modifications. The unmodified construct exhibited a *k*_obs_ of 4 min^−1^. The rate was unchanged when the 14-nucleotide bulged region of the substrate (shown in gray) was replaced by 14 random-sequence nucleotides or when the primer binding site at the 5′ end of the substrate was deleted (constructs not shown). The observed rate was 8 min^−1^ when the 14 bulged nucleotides were replaced by TTTT, but there was no detectable activity (indicated by a dash) when the bulged nucleotides were deleted entirely. A four-nucleotide change to the extended template region of the enzyme resulted in a *k*_obs_ of 0.3 min^−1^, but there was no detectable activity if the corresponding nucleotides in the region of the M3 mutations also were mutated.
(748 KB PDF)
