# Supplementary material for: Darwinian Evolution on a Chip
Source: PLoS Biol. 2008 Apr 8;6(4):e85. doi: 10.1371/journal.pbio.0060085 (PMC2288630; doi:10.1371/journal.pbio.0060085)
Supplement: Supplementary file 1 [file pbio.0060085.sg001.pdf]

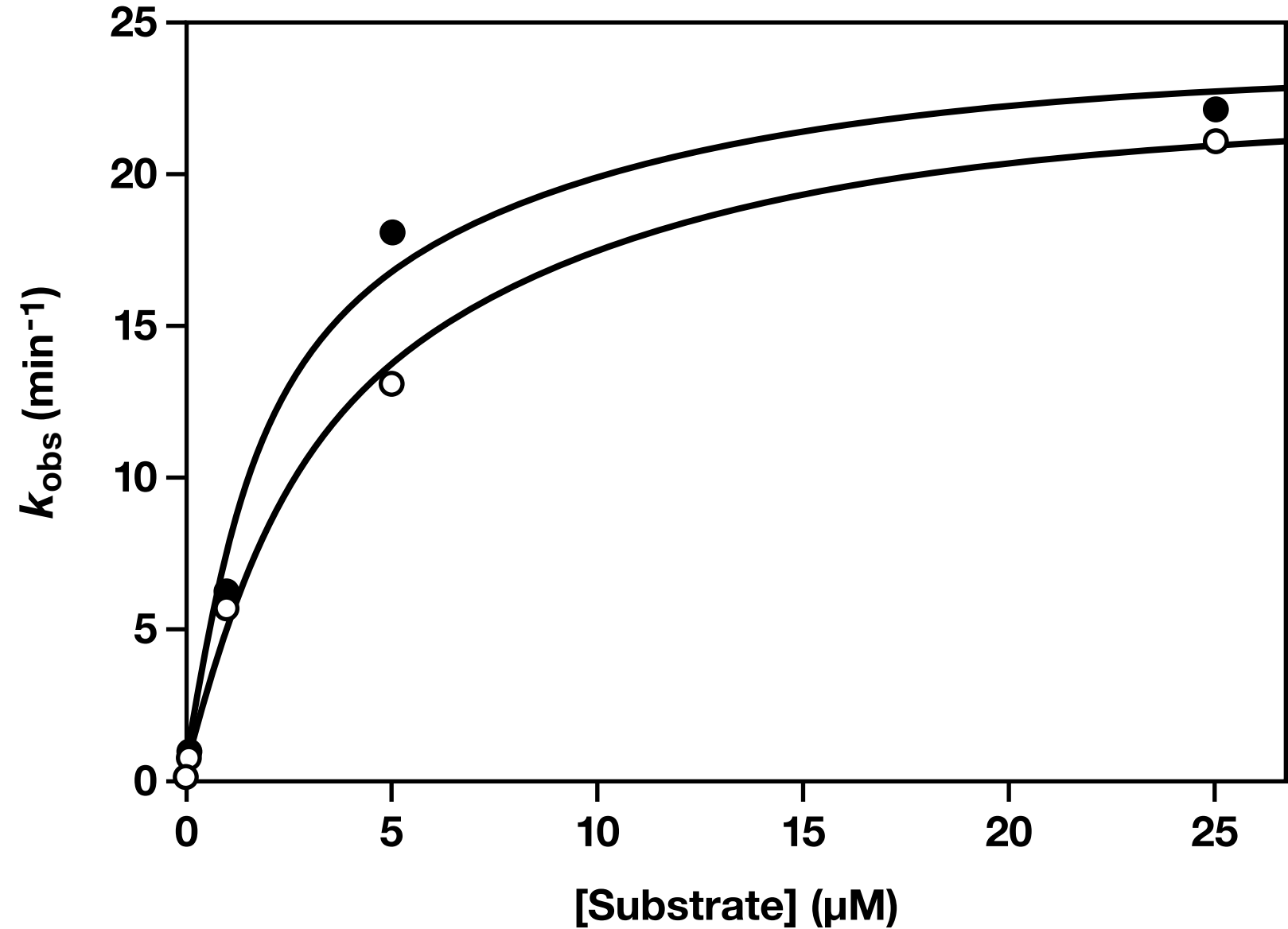

25
20
15
10
5
0
k_{obs} (min^{-1})
0
5
10
15
20
25
[Substrate] (µM)
