# Supplementary material for: Darwinian Evolution on a Chip
Source: PLoS Biol. 2008 Apr 8;6(4):e85. doi: 10.1371/journal.pbio.0060085 (PMC2288630; doi:10.1371/journal.pbio.0060085)
Supplement: Supplementary file 2 [file pbio.0060085.sg002.pdf]

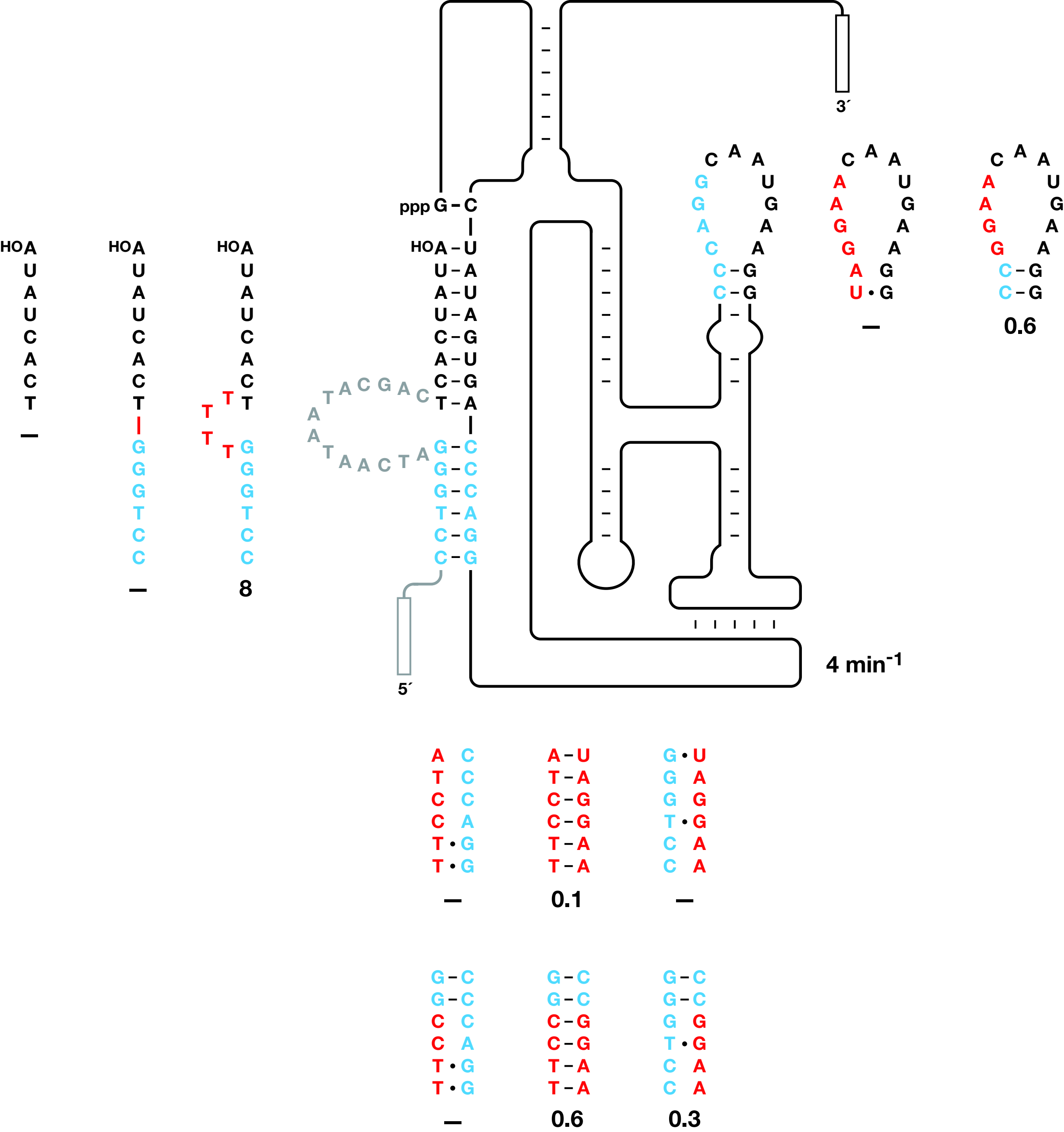

ppp
HO
3´
5´
4 min-1
8
0.6
0.1
0.6
0.3
